# Are the staff in Heddfan Psychiatric Unit, Wrexham Maelor Hospital, are adhering to the personal protective equipment (PPE) guidance as per Public Health Education, England? a QIP

**DOI:** 10.1192/bjo.2021.498

**Published:** 2021-06-18

**Authors:** Asha Dhandapani, Sathyan Soundararajan, Rajvinder Sambhi, Catherine Baker

**Affiliations:** BCUHB

## Abstract

**Aims:**

The aim of this audit is to assess whether healthcare staff are correctly donning and doffing PPE when entering and leaving the wards (changed to donning and doffing PPE when within 2 metres vicinity of a patient).

**Method:**

Consultants/ Junior doctors/ Ward managers/ Staff nurses/ student nurses/ Health care support workers/ Occupational therapist/ Psychologists/ Student nurses/ Housekeeping staff, were all included in this Audit. None of the staff was aware of this Audit and this was an entirely random observation. We used a standard proforma in order to audit. Followed by the Audit, we trained the staff in the unit and then re-audited.

**Result:**

98% of them wore mask whilst in the ward and 94% of them washed their hands after doffing. 36% did not wear them appropriately and about 10-14% did not wear PPE at all. A mere 7 out of 50 alone used hand gel. Overall the donning and doffing of PPE was not being followed and adhered to according to the standards from PHE as per the first Audit. In particular, during donning only 1/3rd of them donned the PPE as per guidance. Likewise, the doffing technique was also poor, with only half of them removing the apron and mask correctly. Unfortunately, only 7 of the 50 people were observed to have used hand gel in between the doffing. This could be potentially increasing the risk of the spread of the coronavirus.

We had trained almost 150 staff members in the Heddfan unit with regard to PPE/ donning and doffing.

Handwashing prior to donning was achieved by all the staff. All the staff, that is 100 % of them adhered to the donning technique in line with the guidance in comparison to just 64% during the first Audit. Whilst hardly just 1/2 to 2/3rd of the staff followed the doffing technique adequately, the second audit showed that only 2 of the 50 staff did not follow the guidance. A meagre/ handful of them followed the utilisation of hand gel in between the tasks of doffing during the first Audit. Almost 90% of them followed the technique properly during the second Audit. Thus showing that the PPE training was successful.

**Conclusion:**

Following the PPE training that was provided to them there was a good response from the staff and this went on to show how effectively we have managed the prevention/ contamination of virus in our unit.

